# The role of oral amoxicillin/clavulanic acid in the treatment of osteomyelitis: a systematic review

**DOI:** 10.1007/s00068-025-03028-w

**Published:** 2026-01-22

**Authors:** Elena Sofia Giovanna Ercolin, Holger Bäthis, Benedikt Marche, Bertil Bouillon, Robin Otchwemah

**Affiliations:** 1 Central Pharmacy, Cologne Merheim Medical Center, University Hospital of Witten/Herdecke, Ostmerheimer Str. 200, Cologne, 51109 Germany; 2 Department of Orthopedics, Trauma Surgery and Sports Medicine, University Witten/Herdecke, Cologne Merheim Medical Center, Ostmerheimer Str. 200, Cologne, 51109 Germany; 3 Institute of Hygiene, Cologne Merheim Medical Center University Hospital Witten/Herdecke , Cologne, Germany; 4 Chair of Hygiene and Environmental Medicine, Faculty of Health / Department of Human Medicine, University Witten/Herdecke, Witten/Herdecke, Germany; 5 Central Department of Hygiene and Environmental Medicine, University Hospital Essen, University of Duisburg-Essen, Essen, Germany; 6 Chair of Trauma Surgery/Orthopedics, Faculty of Health / Departement of Human Medicine, University of Witten/Herdecke, Witten/Herdecke, Germany

**Keywords:** Osteomyelitis, Oral antibiotic treatment, Amoxicillin clavulanic acid, Bone penetration, Pharmacokinetics

## Abstract

**Purpose:**

Osteomyelitis continues to pose significant challenges in clinical management, with no universally accepted treatment guidelines at either national or international level as of 2025. Amoxicillin/clavulanic acid (AMC) is a widely used β-lactam antibiotic known for its safety, tolerability, and broad-spectrum activity against common osteomyelitis pathogens. This work seeks to determine whether oral AMC could serve as a viable and effective option in the management of osteomyelitis based on the current treatment landscape of osteomyelitis.

**Methods:**

Preferred Reporting Items for Systematic Reviews (PRISMA) were applied during the preparation of the article. For the structured search in PubMed^®^, The Cochrane Library, and UpToDate^®^, key research questions were defined and translated into specific search terms. Inclusion and exclusion criteria were defined using the population-intervention-comparison-outcome-study design strategy. All relevant publications available from 1981 to March 2025 were considered.

**Results:**

A total of 1,321 publications were identified. After applying in- and exclusion criteria - supported by automation tools − 104 publications were analysed. The number of primary studies specifically investigating AMC for osteomyelitis is limited. However, available data suggest that AMC is effective and demonstrates non-inferiority compared to standard reference antibiotics. Pharmacokinetic data indicate that prolonged and repeated dosing may enhance distribution into deeper compartments, including bone tissue. Reported adverse effects of AMC are generally mild to moderate, in contrast to alternative agents.

**Conclusion:**

The analysis of the included publications and theoretical considerations on the treatment of osteomyelitis show that surgical debridement followed by long-term administration of AMC can be an effective option for empirical therapy.

## Introduction

The treatment of osteomyelitis is complex and challenging. The classification of osteomyelitis can be approached in a variety of ways. Depending on the aetiology, osteomyelitis can be classified as exogenous (post-traumatic or post-operative) or haematogenous [1]. A further categorisation into acute and chronic can be made based on the course of the disease [2].

A dearth of data exists pertaining to the global epidemiology. In general, men are more frequently affected than women. The risk of developing osteomyelitis increases with age. A rising incidence of the condition has been observed in the United States of America and Germany and is accompanied by a rising prevalence of diabetes mellitus, among other factors [3, 4]. 

Treatment of Osteomyelitis is primarily focused on surgical debridement and in certain cases on replacement of implants accompanied by antibiotic therapy [2]. Initially, antibiotic treatment is administered intravenously followed by an oral administration during the outpatient phase. At present (2025), there is no treatment consensus or guidelines at either the national or international level regarding the most beneficial oral antibiotic substances. Despite the administration of antibiotics and surgical debridement, the reinfection rate remains at approximately 20–30% [5]. The issue has ramifications for the healthcare system due to the rising costs of surgical procedures and pharmaceuticals [3].

Amoxicillin/clavulanic acid (AMC) is the most commonly used β-lactam antibiotic worldwide [6]. AMC is characterised by its safety, well-tolerance and cost-effectiveness as an antibiotic [7]. In general, AMC is effective against a range of gram-positive bacteria, including *Staphylococcus spp.*, *Streptococcus spp.*, *Enterococcus spp.*, and Enterobacterales [8]. Furthermore, the spectrum of activity of AMC also covers the most common osteomyelitis pathogens [9].

Although β-lactam antibiotics are considered to be of significant importance in parenteral therapy, their role in oral therapy is controversial due to poor bone bioavailability and bone penetration [10]. Nevertheless, amoxicillin and AMC are mentioned in guidelines for oral treatment, whereas most of the other β-lactams are not [11–13]. Therefore, the aim of this systematic review is to present the current status of treatment options and to evaluate the possible role of oral amoxicillin/clavulanic acid in the treatment concept of osteomyelitis.

## Materials and methods

**Fig. 1:**
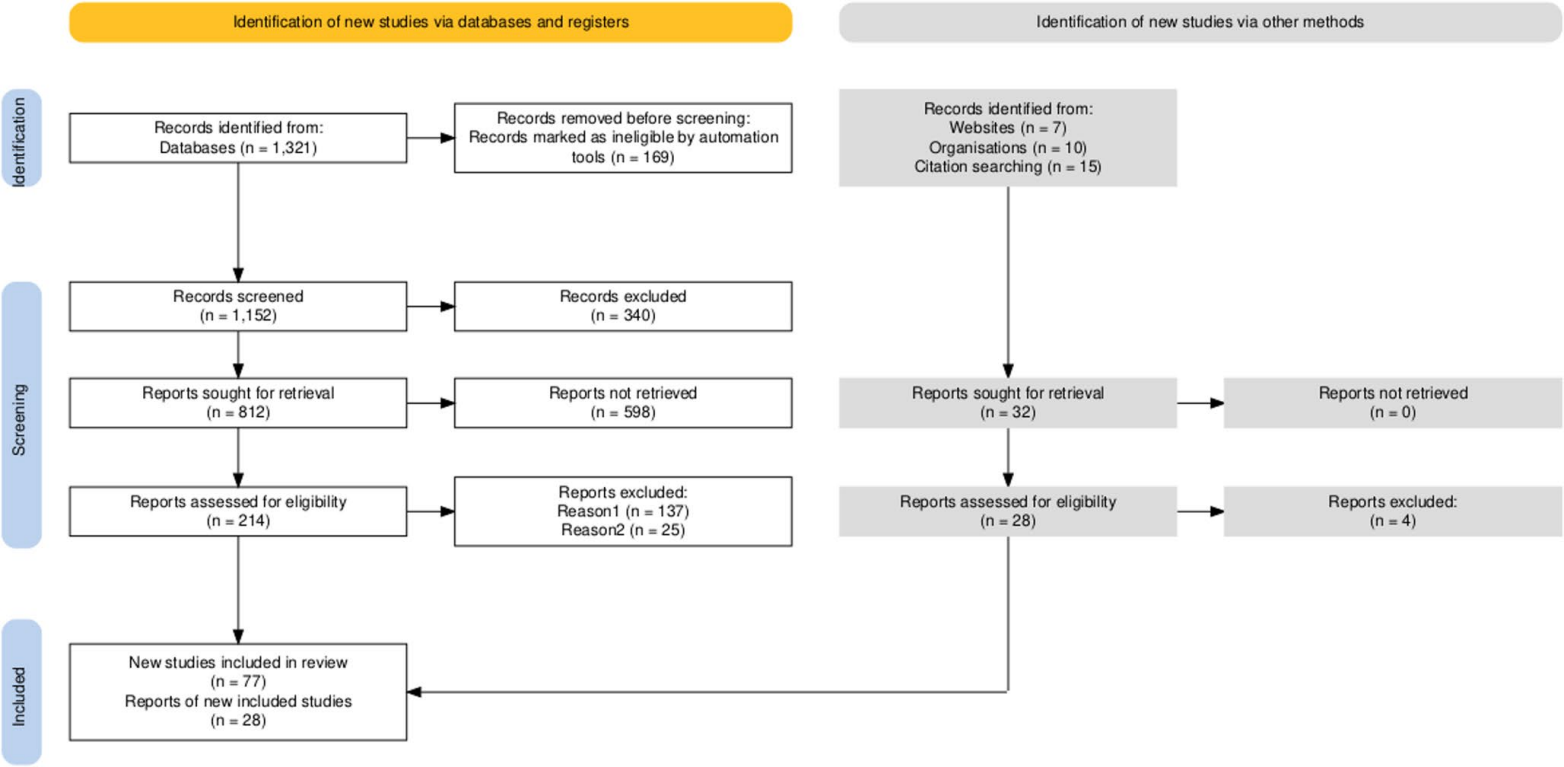
PRISMA flow diagram of screening process for the systematic review of the literature

This systematic review is based on the Preferred Reporting Items for Systematic Reviews and Meta-Analyses (PRISMA) statement guideline [14]. All data were collected in EndNote^™^ 21 and categorized according to the PRISMA checklist. The flowchart of the study evaluation and selection process is shown in Fig. 1 [15].

### Search strategy

The research was conducted in online databases, including PubMed^®^, The Cochrane Library and UpToDate^®^. To ensure that no relevant literature was excluded, the search was supplemented by further articles from additional sources. These were identified by cluster searches from references in the included literature and from the websites of certain national and international professional societies. All search results available up to March 15th, 2025 were considered. The search results include articles published between 1981 and 2025.

The initial stage of the process involved defining key questions to facilitate a systematic approach to the topic. As shown in Table 1, specific search terms were allocated to each key question.

**Table 1:** Allocation of key questions to search terms

Key question	Search term
How is osteomyelitis therapy currently (2024) organised?	• (“Osteomyelitis“[Mesh]) AND “Guideline” [Publication Type] • Osteomyelitis
What is amoxicillin/clavulanic acid and why is it suitable for osteomyelitis therapy? What is the pathogen spectrum? Which pathogen spectrum does amoxicillin/clavulanic acid cover?	• (“Amoxicillin-Potassium Clavulanate Combination“[Mesh]) AND “Osteomyelitis“[Mesh] • ((“Amoxicillin-Potassium Clavulanate Combination“[Mesh]) AND “Treatment Outcome“[Mesh]) AND “Osteomyelitis“[Mesh] • Amoxicillin AND Clavulanic acid AND bone infections • Amoxicillin AND Clavulanate
What is the bioavailability and bone penetration of amoxicillin/clavulanic acid?	• (“Amoxicillin-Potassium Clavulanate Combination“[Mesh]) AND “Biological Availability“[Mesh]
What are the possible adverse effects? What are the advantages of amoxicillin/clavulanic acid over other oral agents? (safety compared to e.g. quinolones, cotrimoxazole, rifampicin)	• Amoxicillin-Potassium Clavulanate Combination/adverse effects [Mesh] • (“Amoxicillin-Potassium Clavulanate Combination“[Mesh]) AND “Drug-Related Side Effects and Adverse Reactions“[Mesh]

The following Medical Subject Headings (MeSH) and keywords were employed: “osteomyelitis”, “bone infection”, “amoxicillin-potassium clavulanate combination”, “guideline”, “treatment outcome”, “adverse effects”, “Drug-Related Side Effects and Adverse Reactions” and “biological availability”. The search terms were combined using the boolean operator “AND”. To clarify the question of safety compared to other substances, the active substance profiles for the corresponding active substances from Uptodate^®^ Lexidrug™ multinational were also included. The same active substance names were used as search terms.

### Study screening

All data were collected in EndNote™ 21 and categorized according to the PRISMA checklist independently by two reviewers. The reviewers engaged in a comprehensive discussion of all identified discrepancies to reach a consensus. Subsequently, the references of the included studies were subjected to a manual search by the reviewers to identify any articles that may have been overlooked during the initial search.

### Inclusion and exclusion criteria

The relevance of the studies was assessed using the population-intervention-comparison-outcome-study design (PICOS) strategy and categorised accordingly [16]. Only studies available in English or German were included in this review.

#### Inclusion criteria

- Population: adults with bone and joint infections.
- Intervention: amoxicillin/clavulanic acid.
- Comparison: placebo, fluoroquinolones, rifampicin, cotrimoxazole, clindamycin.
- Outcome: efficacy including non-inferiority to the compared agents, safety regarding adverse effects.
- Study design: guidelines, systematic reviews, randomised controlled trials, cohort studies and case-control studies.

#### Exclusion criteria

- All non-controlled studies (e.g. case reports/series and letter to the editor).
- Studies focussing on therapies other than antibiotic therapies.
- Studies that were not available as full text.
- Studies that show no benefit for practice.

## Results

### Study selection and study characteristics

A total of 1,321 publications were identified through the literature search conducted in the aforementioned databases and other sources. The use of automation tools resulted in the exclusion of 169 publications. Filters for article language and age were preset in the databases used. Following the application of the established inclusion and exclusion criteria to the titles and abstracts, 245 publications were selected for full-text analysis. Ultimately, 105 publications were included in the review.

### Current osteomyelitis guidelines and treatment recommendations

To summarise current (2025) treatment strategies, 15 publications were identified. The publications comprise guidelines from various nations (France, USA, Japan, Germany and Italy) and reviews. Six publications deal with osteomyelitis in general, without focusing on the site of infection [5, 13, 17–20]. Four publications deal with diabetic foot infections in particular [11, 12, 21, 22]. Three publications focused on vertebral infections [23–25]. One publication each was found on pelvic infections and infections after open fractures [26, 27].

One publication does not provide information on the spectrum of pathogens associated with osteomyelitis [24]. All others describe mainly skin flora such as Staphylococci (e.g. *Staphylococcus aureus (S. aureus)*, including *methicillin-resistant Staphylococcus aureus (MRSA)*) and coagulase-negative Streptococci. Other, generally less common bacteria are gram-negative microorganisms such as Enterobacterales and Pseudomonads [5, 11–13, 17–23, 25–27].

AMC is not mentioned in nine out of 15 publications [5, 17, 18, 20, 22, 23, 25–27]. In the other publications, AMC is mentioned as a treatment option or as an alternative therapy to another first-line therapy [11–13, 19, 21].

Information concerning dosage of AMC is not consistently provided in the available literature. The route of administration is not uniform and varies between oral and parenteral.

Older publications tend to favor parenteral administration because of its better bioavailability [17–19, 24]. In contrast, more recent literature also recommends oral administration [11–13, 21]. 

The International Working Group on the Diabetic Foot (IWGDF) guidance on the diagnosis and management of foot infections in persons with diabetes states that the traditionally parenteral administration and the duration of treatment of at least four weeks is not evidence-based. The guideline proclaims switching to oral therapy after one week is possible if a highly bioavailable antibiotic is chosen. The definition of quality in terms of bioavailability remains unspecified. AMC is not suggested as a treatment option [21]. 

The Infectious Diseases Society of America (IDSA) concurs with this position and states that oral β-lactams should not be utilised for the initial treatment of Native Vertebral Osteomyelitis due to low bioavailability [23]. Another IDSA guideline on diabetic foot infections also refers to parenteral therapy, which should even be continued in the outpatient setting. The advantage would be the better bioavailability of some active substances. The guideline does not specify either the active ingredients or the bioavailability [22]. 

A Japanese guideline postulates that distribution in necrotic bone is low regardless of the active substance. It corresponds to about one tenth of the distribution in blood [17].

An Italian guideline provides a recommendation for the administration of oral AMC in infections caused by *methicillin-susceptible S. aureus (MSSA)*, which is categorised as grade A-II. However, the explanatory text states a grading of B-III, on the grounds that the data available is limited. Oral administration should be considered as sequential therapy. The grading system ranks the strengths of recommendation (A, strongest, to E, weakest) and the quality of evidence I (highest) to III (lowest) [18]. 

2022 German guidelines (expired) provide information on sequential oral therapy. Oral-only therapy is limited to chronic osteomyelitis [19]. 

More recent reviews have focused on the effectiveness of oral therapy. A statistically significant difference between oral and parenteral therapy was not found in the Cochrane Review by Conterno, L. O. et al. on chronic osteomyelitis in 2013 [5]. This contrasts with the UpToDate^®^ recommendation for vertebral osteomyelitis, which suggests empirical parenteral treatment followed by rapid oral switch [25]. Both UpToDate^®^ recommendation on diabetic foot infections and the one on the absence of hardware describe oral regimens under certain conditions. The statements of both recommendations are based on the results of the oral versus intravenous antibiotic for bone and joint infections trial (OVIVA) and individual observational studies. The observational studies did not demonstrate superiority of any specific antibiotic [12, 13]. Two other UpToDate^®^ recommendations concerning open fractures and pelvic osteomyelitis do not specify the route of administration [26, 27]. Neither the reviews nor the OVIVA trail include any reference to oral AMC.

### Amoxicillin/clavulanic acid and osteomyelitis therapy

Regarding the primary literature related to the therapeutic use of AMC in the management of osteomyelitis, eleven relevant articles were identified. Table 2 shows the number of publications in correlation to the key infection characteristics identified in the studies. The analysis of the publications was conducted with a particular emphasis on the following aspects: microorganisms, route of administration, site of infection and the resulting outcome.

**Table 2:** Reporting frequency of key infection

Infection characteristics/author		Route of administration				Microorganism						Site of infection				Outcome			
		Oral	Parenteral	Sequential (parenteral → oral)	Not defined	Gram-positiv	*S. aureus*	*S. epidermidis*	*Gram-negativ*	Anaerobic	Not definded	Diabetic foot infections	Vertebral infection	Bone and joint infections	Not definded	Efficacy of AMC	Bone penetration	Sensitivity of AMC	Other
1	Gariani, K. et al.	x				x	x		x			x				x			
2	Park, K. et al.	x				x	x		x				x					x	
3	Albert, H. B. et al.	x									x		x						x
4	Landerdorfer, C.B. et al.		x			x	x	x							x		x		
5	Landerdorfer, C.B. et al.		x								x		x				x		
6	Weumaier, K.		x						x					x			x		
7	Patel, S. et al.				x						x				x			x	
8	Lipsky, B. A. et al.			x		x			x			x				x			
9	Gisby, J. et al.				x	x									x	x			
10	Walter, G. et al.				x	x			x	x				x			x		
11	Armstrong, D. G. et al.				x	x	x	x							x				x

#### Microorganisms

A total of eight publications refer to microorganisms associated with osteomyelitis, while the remaining three do not describe any microorganisms. Seven publications refer to gram-positive microorganisms, five include gram-negative microorganisms additionally or exclusively, and one publication focuses on anaerobic microorganisms. *S. aureus* is mentioned as an osteomyelitis-associated pathogen in five publications [28–32].

Two of these articles explicitly differentiate between *MSSA* and *MRSA*. Methicillin-susceptible strains are more common than methicillin-resistant strains [28, 29]. 

Further gram-positive, aerobic pathogens include Coagulase-negative Staphylococci. Two publications also refer specifically to *Staphylococcus epidermidis (S. epidermidis)* [31, 32]. Gram-negative microorganisms are mentioned secondarily. Anaerobic bacteria can be characterised as atypical for osteomyelitis [33].

#### Route of administration

Three articles each specify an oral or parenteral application [28, 29, 31, 34–36]. One publication refers to parenteral application followed by oral therapy [37]. In four cases, the route of administration is not defined [30, 32, 33, 38]. One of these publications reports on the results of an animal trial [30].

#### Sites of infection

In the included literature, details of the localisation of infection are heterogeneous. Two publications address diabetic foot infections [28, 37], three focus on vertebral osteomyelitis [29, 34, 35], two address bone infections, including joint infections [33, 36], and four deal with infections of unspecified location [30–32, 38].

#### Outcome

Three of the publications describe the efficacy of AMC in context of osteomyelitis therapy. Gariani, K. et al. describe a remission rate of oral AMC on par with non-ß-lactam-based oral regimens (e.g. quinolones, glycopeptides, clindamycin, cotrimoxazole or rifampicin) [28]. Remission was observed in 74% (*p* = 0.15, CI = 0.5–1.6) of 419 patients suffering mainly from diabetic foot infections or osteomyelitis caused by *Enterobacteriaceae* (64,2%) or *MSSA* (50,6%) after treatment with oral AMC. In comparison, non- β-lactam regimens show a remission in 79% of the cases. Intraoperative samples frequently exhibited evidence of mixed bacterial colonization [28].

Comparable remission rates of AMC (83%) compared to quinolones (85%) were also shown in the study by Lipsky, B.A. et al. [37]. In the study, both regimes were administered in sequence: initially parenterally, and subsequently orally [37].

Gisby, J. et al. demonstrated non-inferiority of AMC to flucloxacillin and clindamycin in an animal model [30]. After subcutaneous administration of amoxicillin, clavulanic acid and flucloxacillin, the concentrations in infected bone were lower than in serum. The penetration percentage ranged between 10 and 21% for all substances tested [30]. 

Park, K. et al. refer to the sensitivity of the bacteria described to AMC [29]. Nevertheless, no evidence is provided to demonstrate the actual efficacy of the substance [29]. 

Patel, S. et al. only stated a theoretical effectiveness of AMC, no evidence in the form of clinical trials was provided [38].

A detailed description of bone penetration and distribution between the bone tissues can be found in the work of Landersdorfer, C.B. et al. and Weismeier, K. et al. [31, 36]. Parenteral administration was used in both studies [31, 36]. The concentration of amoxicillin and clavulanic acid in cortical and cancellous bone is lower than in serum [31]. It was observed that the two substances demonstrated slightly greater levels of penetration into the cortical bone in comparison to the cancellous bone [36]. The median of the ratio of the area under the curve (AUC) for bone/AUC for serum was 20% in cortical bone and 18% in cancellous bone for amoxicillin and 15% in cortical bone and 10% in cancellous bone for clavulanic acid [31].

The study by Walter G. et al. is based on the results of Landersdorfer C.B. et al. 16% of the included patients received AMC. Other antibiotics used in this trial were Rifampicin (33%), Vancomycin (31%), Quinolone (31%), Amoxicillin (30%), Metronidazole (30%), Clindamycin (26%), Cotrimoxazole (23%), Imipenem/cilastatin (18%), Ceftriaxone (15%), Doxycycline (7%), Aminoside (5%), Piperacillin–tazobactam (3%) and Fusidic acid (3%). The study did not provide data on the remission rate for the individual substances. An overall remission rate of 77% was reported [33]. 

Housden P.L. et al. examined bone penetration of AMC in the context of perioperative, parenteral prophylaxis: Compared to Cefuroxime, AMC bone penetration is reported to be marginally inferior [35].

Two further studies focus on other aspects related to the outcome of AMC in osteomyelitis therapy. Albert H. et al. hypothesised that lower back pain could be associated with bacterial infections. The administration of oral AMC is stated to have clinical effects [34].

Regarding the prevalence of methicillin resistance, Armstrong D.G. et al. compared the occurrence of methicillin resistance between ciprofloxacin and AMC after long-term administration (>2 weeks) [32].

### Bioavailability

In addition to the studies listed before on bone permeability, three further studies on bioavailability were retrieved. None of the publications provide data on bone penetration. Instead, the oral bioavailability and the influence of food intake are analysed.

De Velde, F. et al. provide a comprehensive description of the pharmacokinetics of clavulanic acid [39]. The morning doses demonstrated higher maximum plasma concentration (C_max_) and area under the curve from time 0 to 8 (AUC_0 − 8_) values in the two-thirds case in comparison to the subsequent doses. After oral administration, the bioavailability is variable and depends on the amoxicillin dose [39]. 

In the study of Weitschies, W. et al. and Staniforth, D. H. et al. the dependence of bioavailability on food intake is investigated and both demonstrate contradictory results [40, 41]. Weitschies, W. et al. found a reduced bioavailability of clavulanic acid after food intake [40]. Conversely, amoxicillin shows increased bioavailability after food intake [40]. Moreover Staniforth, D. H. et al. concluded that the bioavailability is not influenced by food intake [41]. Rather, they found that food intake has a positive effect on possible side effects such as nausea and vomiting [41].

### Adverse reactions of AMC in contrast to other antibiotics relevant for therapy

**Fig. 2:**
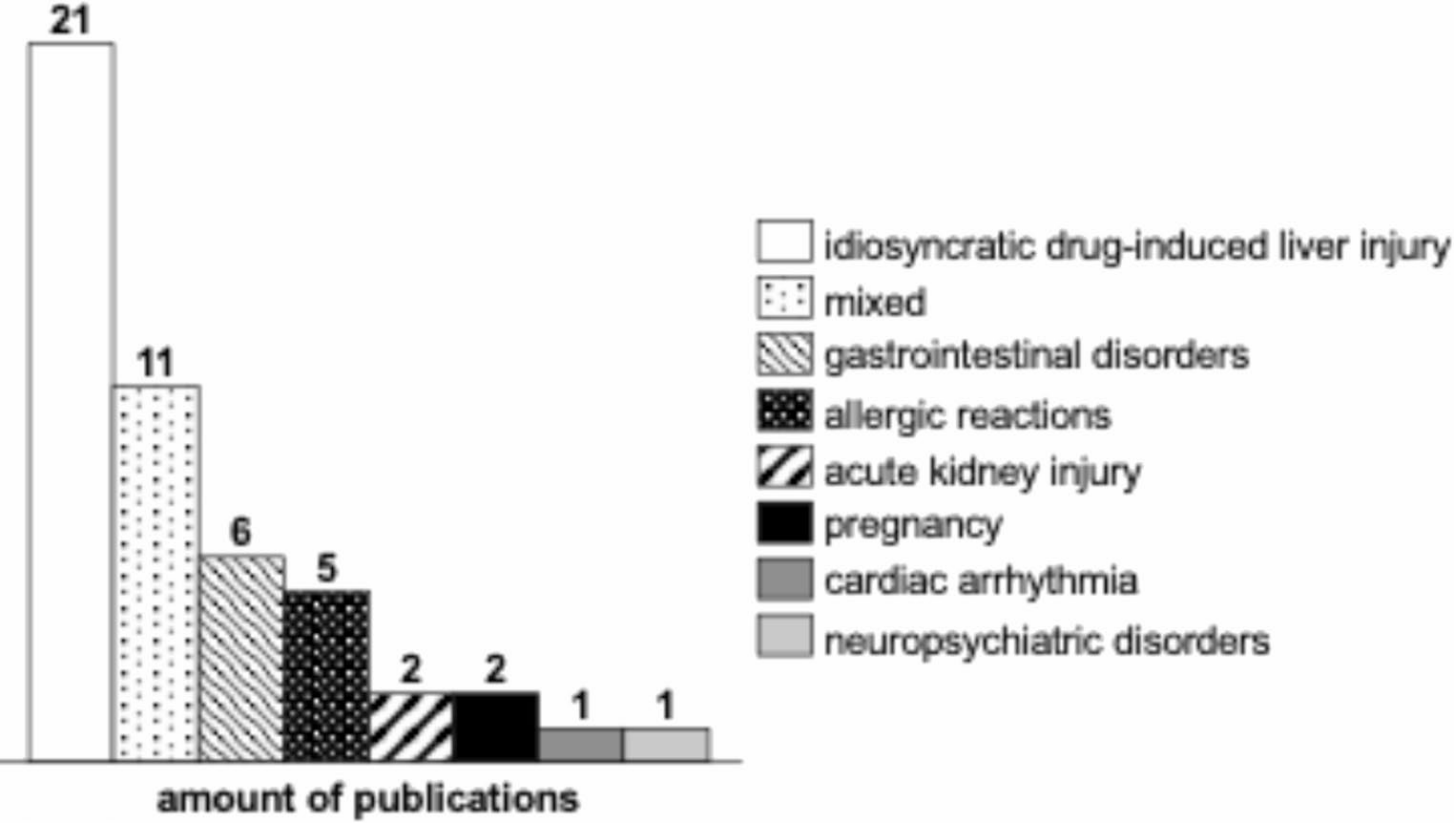
Distribution of adverse effects among the included publications

Based on the two MeSH-terms relating to adverse effects, 119 reports were identified as eligible. Of these, 49 were ultimately included in this review. 28 papers compared the adverse reactions of AMC to other drugs. The adverse reactions described in the publications can be grouped into eight categories. The categories identified and their frequency are shown in Fig. 2. The majority of publications are not able to establish a correlation between the administered doses and the subsequent occurrence of adverse effects, both in terms of their frequency and intensity. Only five publications offer insights into a potential dose-dependency. Two cases were observed in connection with drug-induced liver injury (DILI) and one case each with regard to gastrointestinal complaints, to nephropathy and to adverse effects in general. The following section delineates the individual side effects.

#### Drug-induced liver injury (DILI)

The most frequently investigated side effect is that of DILI (Fig. 2). Associated symptoms include jaundice, pruritus, fatigue and decreased appetite [42–51]. Laboratory tests typically reveal an elevation in liver enzymes, with cholestatic changes being the most prevalent [42, 44, 48–50, 52–57]. These alterations demonstrate a tendency towards increased prevalence in the elderly population and in males [42, 44, 47, 48, 57, 58].

Contrary to this, Lucena, M.I. et al. did not observe a increased risk for males [57]. Gu, S. et al., Tailor, A. et al., Hunt, C. M. and O’Donohue, J. et al. delineate a genetic HLA-associated predisposition that favours the occurrence of DILI [46, 55, 59, 60].

In an animal study on liver toxicity, a dose-dependent relationship was demonstrated for the opening of mitochondrial membrane pores, which is associated with the malfunctionality of liver cells [61].

As Thomson, J. A. et al. reiterate, there is an absence of a substantial correlation between the incidence of jaundice and AMC dose [47].

The latency period between the initiation of therapy and the onset of liver failure demonstrates variability, ranging from a week to a maximum of three months [42, 46, 48, 60, 62]. However, it has been observed that the latency period undergoes a reduction in duration following repeated exposure [55]. The severity of adverse effects is illustrated by Suzuki, A. et al., Lucena, M. I. et al. and Andrade, R. J. et al. [45, 54, 56, 57]. In the case of hepatocellular manifestations of DILI, the course is usually more severe and can lead to necessity of transplantation or death [45, 56]. In the majority of cases, a cholestatic manifestation is observed [42, 48–50, 54, 56–59]. The results of Lucena, M. I. et al. indicate a probability of 2.9% for the occurrence of the most fatal outcome for patients who developed DILI [57]. Furthermore, a higher risk is associated with the simultaneous intake of other hepatotoxic substances [54]. Tailor, A. et al., O’Donohue, J. et al. and Wong, F. S. et al. stated that clavulanic acid is primarily responsible for the occurrence of DILI [46, 49, 60].

17 of the publications do not specify the route of administration, while two specify oral administration and intraperitoneal administration in the context of an animal study. A single publication reports on both oral and parenteral administration, yet no information is provided on potential disparities in severity of DILI [58].

#### Gastrointestinal disorders

In all the studies on gastrointestinal disorders, diarrhea is reported as the main symptom [41, 63–67]. Caron, F. et al. and Staniforth, D. H. et al. mentioned nausea and vomiting [41, 67]. The study by Bax, R. concludes that the gastrointestinal effects can be reduced by limiting the dose from three times a day to twice a day [64]. This finding is concomitant with the observation made by Caron, F. et al. that the oral administration of AMC in therapeutic doses exerts a significant effect on the motility of the duodenum-jejunum in human subjects [67]. It is postulated that clavulanic acid is the main cause of the gastrointestinal disorders [64]. The animal study by Spiro, D. M. et al. also provides evidence that AMC is associated with mesenteric adenopathy [65]. However, there is an absence of evidence in the study by Spiro, D. M. et al. to support this hypothesis in human cohorts [65].

#### Acute kidney injury (AKI)

Thomas, L et al. describe Amoxicillin-associated crystalluria (AICN) by employing the French national pharmacovigilance database [68]. AICN is categorised as a “known but rarely reported adverse reaction” of AMC [68]. The risk of AICN is associated with high doses and rapid administration. The phenomenon occurs more frequently with parenteral application [68]. 

In the comparative study conducted by Walker, H. et al., AMC was compared to the combination of flucloxacillin and gentamicin [69]. It was found that AMC is associated with a reduced rate of postoperative AKI. The route of administration was not defined [69].

#### Allergic reactions

The study by Freundt-Serpa, N. P. et al. characterises the occurrence of acute and delayed allergic reactions to AMC [70]. The importance of clavulanic acid is also highlighted. A confirmed allergic reaction was documented in a minority of the study population. An allergy to clavulanic acid identified by skin testing has no diagnostic advantage [70]. 

The symptoms of the allergic reaction have been defined by Freundt-Serpa, N. P. et al. and Confino-Cohen, R. et al. as urticarial rash and, in rare cases, anaphylactic shock [70, 71]. Both studies consider skin tests to be useful predictors for the rational use of AMC and the avoidance of allergic reactions [70, 71].

The publication by Tee, C.T. et al. provides an analysis of severe cutaneous adverse reactions [72]. The overall incidence caused by the investigated drugs is minimal. AMC was most frequently associated with acute generalised exanthematous pustulosis (AGEP) [72]. 

In addition to the allergic manifestations previously documented, two further phenomena were identified, which can be classified as allergic in the broadest sense. In the case of drug-induced aseptic meningitis triggered by AMC, delayed type 4 hypersensitivity or T-cell mediated hypersensitivity is assumed. The study demonstrates a predominance of male subjects among the affected population [73]. 

In their analysis of two global pharmacovigilance databases (EudraVigilance and VigiLyze), Renda et al. found a connection between AMC and Kounis syndrome [74]. Kounis syndrome is also attributable to anaphylaxis or, in general, to hypersensitivity. In this case, however, a cardiac manifestation is observed [74]. None of the available studies specified the route of administration.

#### Pregnancy

Two studies on the safety of AMC in pregnancy were identified. Both confirm the safe use of AMC and indicate no increased risk of congenital malformations [75, 76]. As demonstrated by the findings of one of the two studies, this is also confirmed for the administration of the substance orally [76].

#### Cardiac arrhythmia

The study by Chou, H. W. et al. compares the risk of cardiac arrhythmia of oral AMC to macrolids and fluoroquinolones [77]. In particular, azithromycin and moxifloxacin carry a significant risk of ventricular arrhythmia and cardiovascular death compared to AMC [77].

#### Neuropsychiatric disorders

Zhang et al. undertake a comparative analysis of the potential for neuropsychiatric disorders associated with the use of fluoroquinolones and AMC [78]. The findings of this study indicate that there is a comparable risk of such adverse effects occurring with both substances [78]. The route of administration was not defined.

#### Mixed

Ten of the publications provide an overview of the possible side effects of AMC. The side effects include gastrointestinal disorders, hepatotoxicity and allergic reactions [79–87]. Only Salvo, F. et al. describe hematological changes identified as purpura [88]. The severity of the adverse effects is generally categorised as mild to moderate [80, 85].

With increasing doses of clavulanic acid alone, a dose-dependency for adverse effects has been reported. It is also possible to observe mild to moderate severity in this case [80]. 

Of the publications under review, five refer to the oral administration [79, 80, 84, 86, 89]. Two studies have been conducted that consider parenteral followed by oral therapy [85, 87]. Four of the studies analysed did not provide any information on the route of administration [81–83, 88].

A total of 12 articles compared adverse reactions caused by AMC with those of other antibiotics. As active substances or classes relevant for osteomyelitis clindamycin, cotrimoxazole, fluoroquinolones, macrolides and rifampicin can be identified.

Gastrointestinal side effects such as diarrhea or pseudomembranous enterocolitis are also reported for clindamycin. The latter is to be regarded as a serious side effect [81]. Compared to AMC, headaches occur more frequently with clindamycin [80].

Cotrimoxazole has been observed to induce a relatively high rate of allergic reactions and haematological changes. These manifestations have been found to be more pronounced in comparison to those observed with AMC [81, 83]. 

In the comparative studies, ciprofloxacin and ofloxacin were the predominant fluoroquinolones utilised. Adverse effects, including gastrointestinal symptoms and hepatotoxicity, have been reported in conjunction with these medications. The hepatotoxic effect has an earlier onset when fluoroquinolones are used and occurs less frequently in comparison with AMC. However, DILI with fluoroquinolones tend to be more severe [59, 61, 84, 85]. In contrast to AMC, the occurrence of neuropsychiatric changes, such as headaches and dizziness, as well as tendon ruptures and phototoxicity, has also been documented [78]. Comparing moxifloxacin versus piperacillin/tazobactam followed by AMC, mild to moderate side effect intensities were reported for all substances. The most frequently reported adverse effects were diarrhea and nausea. A differentiation of the substances in the control group with regard to side effects was not observed [89]. Regarding the occurrence of methicillin resistance a higher probability was postulated in cases where previous therapy involved the administration of ciprofloxacin. Long-term treatment (>2 weeks) with ciprofloxacin was used in 54% of cases in which methicillin-resistant coagulase-negative staphylococci were detected. The occurrence of MRSA selection was observed in 23% of cases treated with oral AMC in the preceding period [32]. 

## Discussion

To assess the role of oral AMC in osteomyelitis therapy, it is necessary to consider and categorise the results concerning efficacy and safety in a synergistic manner. In consideration of the spectrum of activity of AMC and the microorganisms associated with osteomyelitis, the existing literature can substantiate a possible relevance for use in therapy [11–13, 19, 21, 28, 30, 37]. The most common bacteria that can be isolated in the presence of osteomyelitis are widely susceptible to AMC [9, 28–32].

In accordance with the clinical breakpoints established by the European Committee on Antimicrobial Susceptibility Testing (EUCAST), minimal inhibitory concentrations (MICs) of 0.03–0.5 mg/L are designated for AMC in the treatment of *S. aureus*, and 0.001 mg/L for Enterobacterales and 4 mg/L for Enterococci. The MICs are based on oral dosages for AMC of 500 mg/125 mg three times a day for the low dose and 875 mg/125 mg (also defined as 1 g AMC) thrice daily for the high dose [8, 90]. 

It is posited that, in consideration of the pharmacokinetic and pharmacodynamic properties of β-lactam antibiotics, a time-dependent mode of action is defined. It is imperative to ascertain that the dosing interval, or the percentage of the dosing interval in which the plasma concentration is above the MIC of the pathogen (t >MIC or %t >MIC), is attained [19]. The objective for AMC is to ensure a time of the unbound fraction (fT) >MIC value of at least 40–50% of the duration of the dosage interval [91].

The current breakpoints for Enterococci are related to parenteral application. In the case of oral administration, the only data available pertains to urinary tract infections. Common doses of parenteral AMC were able to achieve target values of 40% fT >MIC for MICs up to 4 mg/L. The 50% target could be achieved with a daily dose of 4 g of AMC [90]. 

Furthermore, the bioavailability of AMC must be considered when evaluating its efficacy. Oral bioavailability of amoxicillin in serum ranges from 72 to 94%, while that of clavulanic acid is 60% [19]. It is important to note that the oral bioavailability is expressed as a percentage, assuming 100% bioavailability after parenteral administration.

The study by Landersdorfer, C.B. et al. confirms this assumption even though the administration of AMC has been also parenterally. Contrary to the recommendations of EUCAST, a single dose of 2000 mg/200 mg of AMC was administered. AMC administered every four hours has shown to achieve robust (>90%) probabilities of target attainment (PTAs) for MICs of < 12 mg/L in serum and 2 to 3 mg/L in bone and population. Furthermore, population PTAs above 95% have been demonstrated against MSSA in bone and serum [31]. 

Considering the oral bioavailability in serum, a dosage of at least 6 g daily or more would also have to be administered orally in order to achieve 40–50% (fT) > MIC. According to the EUCAST, the daily application of 3 g of AMC is the standard procedure. In consideration of the bioavailability previously delineated, the therapeutic intervention may be administered in a suboptimal dosage. Nevertheless, the persistence of clinical successes suggests that surgical interventions are likely to be a contributing factor.

Although a study on atypical pathogens for osteomyelitis could be identified, the efficacy of AMC cannot be proven [33]. The quantity of primary literature in which oral AMC was employed to treat osteomyelitis is limited. Nevertheless, efficacy and non-inferiority to the reference substances can be deduced from the available results As evidenced by the data those studies indicated that AMC, when administered orally, resulted in remission in comparison to among other quinolones [28, 30, 37].

In order to assess the efficacy of AMC at the site of action, it is necessary to consider the issue of bone penetration, which is prerequisite. The included studies on the bone permeability of AMC show strongly diverging statements [31, 36, 92]. The special tissue properties of bone must be taken into account when analysing bone penetration. Bone is partially poorly perfused tissue and can be divided morphologically into cancellous bone and cortex. Each exhibits different perfusion characteristics [93]. The question of which tissue is the predisposed habitat of pathogens remains unresolved. It is generally assumed that most pathogens are located in the interstitium [19].

It can be hypothesised that bacterial proliferation is most likely to occur within the organic or aqueous components of bone tissue [92]. Amoxicillin (LogP = −2.31) and clavulanic acid (LogP = −1.52) are both deemed to be hydrophilic substances, based on their partition coefficient [94, 95]. 

Consequently, it can be deduced that an accumulation of these substances also occurs preferentially in a hydrophilic environment. However, the prediction of the absorption of these substances by other components, such as calcium salts or hydroxyapatite, within the bone is challenging.

Previous studies have investigated the absorption of β-lactams into inorganic bone components. The results obtained demonstrate a low binding capacity. Instead, an extracellular and interstitial distribution can be observed [31, 36]. Nonetheless, given the established bioavailability following oral administration and the considered studies, the likelihood of low absorption is presumed.

In general, the available data on this subject is limited due to the necessity of in vivo testing, which invariably involves a bone biopsy. All studies regarding bone penetration were conducted with parenteral administration of AMC.

Including the oral versus intravenous antibiotic for bone and joint infections trail (OVIVA), which suggests the equivalence of oral and parenteral administration, an analogy with bone bioavailability after oral administration can also be assumed, even if an outcome by treatment with AMC is not explicitly mentioned [96].

The Major Extremity Trauma Research Consortium (METRC) study presents similar results. The study protocol also includes the possibility of treatment with AMC for *Enterococci* and *Bacteroides spp*. The reporting of specific results for AMC is not a part of this research [97]. Furthermore, parenteral administration is associated with additional risks, such as catheter associated sepsis and increased costs [97].

All publications concerning bioavailability administered AMC in the perioperative setting, so that the duration of application before testing was relatively brief [31, 36]. It is evident that under these circumstances, the achievement of a steady-state concentration is not achievable. The treatment of osteomyelitis is usually of a duration of several weeks [19].

The half-life of AMC is approximately 1–1.5 h [9]. The redistribution of AMC from the serum into the less accessible compartment of the bone is required to occur rapidly in order to achieve a constant concentration in bone. Pharmacokinetically, prolonged and repeated exposure to the drug can be expected to result in increased distribution to deeper compartments such as bone.

Two reviews on the bioavailability of various antibiotics in bone additionally confirm the bone penetration of AMC, although mostly parenteral application and different dosages were considered [92, 98].

In addition, the data reported by Thabit, A.K. et al. is compared with the MIC of osteomyelitis-relevant bacteria. Data is available for Enterococci, β-hemolytic Streptococcus *(S. pyogenes)*, Viridans group Streptococcus, Enterobacteriacae and Anerobes. It is hypothesised that for the treatment of these pathogens AMC demonstrates robust penetration capabilities despite the presence of a concentration ratio of 10:1 in the serum to bone [98]. 

With regard to the safety of AMC, the spectrum of side effects were shown in Fig. 2.

The most prevalent adverse effects were DILI, gastrointestinal complaints and allergic reactions [41–67, 70–74]. Acute kidney injury, neuropsychiatric disorders and cardiac arrhythmia were classified as secondary [68, 69, 77, 78]. In the studies conducted on the latter phenomena, there is no available data on the severity of the events that occur. In view of the aforementioned factors, in conjunction with the infrequency of the occurrence, the utilisation of AMC should not be prematurely dismissed in the risk-benefit assessment. In regard to the duration of treatment for osteomyelitis, consideration should be given to the monitoring of these adverse effects.

In the context of DILI, both mild to moderate courses, but also severe forms are documented. Although severe courses are rare, liver function in particular should be monitored. Predisposing factors such as male gender, age and previous DILI under AMC should be taken into account in the extent of monitoring. In order to reach a decision regarding the administration of AMC therapy, it is necessary to consider concomitant diseases and medication, which have also been demonstrated to promote DILI [42, 44, 45, 47, 48, 54, 56–58]. 

Contrary to the commonly accepted assumption that the hepatotoxic effect is more pronounced with parenteral AMC administration compared to oral administration, the available data did not support this hypothesis, as there is an absence of any correlation between the severity of DILI and the route of administration. The assumption under discussion is based on the theoretical hypothesis that clavulanic acid is primarily responsible for the hepatotoxicity. The bioavailability is reduced when administered orally. It can be hypothesised that a correlation exists between bioavailability and hepatotoxicity.

Allergic reactions attributable to AMC should be critically scrutinised due to the frequent misdiagnosis of a penicillin allergy. The existing literature further demonstrates that instances of severe anaphylaxis, AGEP, Steven-Johnson syndrome, and toxic epidermal necrolysis are exceedingly rare. Conversely, penicillin allergy delabelling should be conducted prior to the initiation of therapy, favouring an otherwise well-tolerated therapy with AMC [70–72]. 

The severity of other hypersensitivity reactions, such as aseptic meningitis and Kounis syndrome, should not be underestimated. However, these reactions are very rare, and thus even this potential side effect does not negate AMC as a treatment option [73, 74]. 

Gastrointestinal complaints are common with AMC. Symptoms are mild to moderate. However, due to the long duration of treatment for osteomyelitis, persistent diarrhoea, nausea and vomiting can be treatment-limiting [41, 63–67]. 

For AMC, the following side effects are reported as significant in the Lexi-Drug^®^ multinational: gastrointestinal side effects including Clostridioides difficile infection, DILI and allergic reactions including drug-induced enterocolitis syndrome. The latter was not mentioned in any of the publications reviewed. The other adverse reactions are consistent with the findings above [99]. 

These significant side effects are to be compared in the following with the other treatment options. Gastrointestinal events, including diarrhoea, nausea and vomiting, have also been reported in association with treatment using clindamycin and fluoroquinolones [100, 101]. Clindamycin can also cause the serious side effect of pseudomembranous colitis. A comparison of the incidence of Clostridium difficile infections reveals that it is higher with clindamycin and equivalent for cotrimoxazole, fluoroquinolones and rifampicin when compared with AMC [102, 103].

The hepatotoxic potential of cotrimoxazole, fluoroquinolones and rifampicin has been documented [101, 104, 105]. Fluoroquinolones have been shown to induce hepatotoxicity with a reduced frequency and earlier onset compared to AMC [99, 101]. However, the cases reported are typically more severe. Allergic reactions have also been reported in association with alternative therapy options. Cotrimoxazole and rifampicin have been observed to induce adverse effects that include alterations in complete blood counts [104, 105]. For the fluoroquinolones in particular, a significantly higher prevalence of adverse effects of a certain severity is documented [101]. With regard to the psychiatric effects of AMC, the equivalence to ciprofloxacin could only be proven with one source [78]. Consequently, it is not pertinent to consider this aspect when determining a course of treatment for AMC.

The treatment alternatives possess a greater propensity for the occurrence of adverse effects, exhibiting a similar range of side effects. Moreover, alternative substances have been demonstrated to be associated with the emergence of additional side effects that manifest with greater frequency and are characterised by a more severe nature. Fluoroquinolones require additional ECG monitoring during treatment to detect the occurrence of QT prolongation. Co-medication, which also carries a risk of QT prolongation, must also be taken into account [101].

In comparison to AMC, such fundamental monitoring measures are not deemed necessary. The utilisation of AMC should be contemplated on a risk-adapted foundation, with consideration given to prevailing restrictions.

In circumstances where higher dosages might be necessary to attain adequate bone penetration, it could be deduced that safety is equally guaranteed in such cases.

The data currently available on standardised therapies with AMC is limited. These show a gap for the treatment of long bones. The focus of the publications is on diabetic infections and vertebral osteomyelitis. Information regarding dosage is rarely reported in the publications, and the recommendations provided vary significantly, thus no dosage recommendation can be derived.

## Conclusion

In conclusion, the results of the trial and the theoretical considerations on the efficacy and safety of AMC in the treatment of osteomyelitis support the hypothesis but cannot fully substantiate it. Of particular concern is the issue of bone permeability and the subsequent bioavailability of the substance in the bone following oral administration, both of which remain uncertain. Notwithstanding the demonstration of clinical success, it can be hypothesised that this will be supported by additional surgical debridement and long-term application. This finding may be of particular importance for empirical therapy, as oral AMC represents an economical and safe therapeutic option. In addition, shorter hospitalisations can be achieved through oral administration. Nevertheless, an observational study with an appropriate study design is necessary to verify the hypothesis.

## Data Availability

No datasets were generated or analysed during the current study.
